# GTPase Activating Protein (Sh3 Domain) Binding Protein 1 Regulates the Processing of MicroRNA-1 during Cardiac Hypertrophy

**DOI:** 10.1371/journal.pone.0145112

**Published:** 2015-12-16

**Authors:** Minzhen He, Zhi Yang, Maha Abdellatif, Danish Sayed

**Affiliations:** Cardiovascular Research Institute, Department of Cell Biology and Molecular Medicine, Rutgers New Jersey Medical School, Newark, New Jersey, United States of America; IRCCS-Policlinico San Donato, ITALY

## Abstract

**Background:**

MicroRNAs (miR) are small, posttranscriptional regulators, expressed as part of a longer primary transcript, following which they undergo nuclear and cytoplasmic processing by Drosha and Dicer, respectively, to form the functional mature ~20mer that gets incorporated into the silencing complex. Others and we have shown that mature miR-1 levels decrease with pressure-induced cardiac hypertrophy, however, there is little or no change in the primary transcript encompassing miR-1 stem-loop, suggesting critical regulatory step in microRNA processing. The objective of this study was to investigate the underlying mechanisms regulating miR-1 expression in cardiomyocytes.

**Results:**

Here we report that GTPase–activating protein (SH3 domain) binding protein 1 (G3bp1), an endoribonuclease regulates miR-1 processing in cardiomyocytes. G3bp1 is upregulated during cardiac hypertrophy and restricts miR-1 processing by binding to its consensus sequence in the pre-miR-1-2 stem-loop. In accordance, exogenous G3bp1 is sufficient to reduce miR-1 levels, along with derepression of miR-1 targets; General transcription factor IIB (Gtf2b), cyclin dependent factor 9 (Cdk9) and eukaryotic initiation factor 4E (Eif4e). While Cdk9 and Gtf2b are essential for transcription, Eif4e is required for translation. Thus, downregulation of miR-1 is necessary for increase in these molecules. Similar to miR-1 knockdown, G3bp1 overexpression is not sufficient for development of cardiac hypertrophy. Conversely, knockdown of G3bp1 in hypertrophying cardiomyocytes inhibited downregulation of miR-1 and upregulation of its targets along with restricted hypertrophy, suggesting that G3bp1 is necessary for development of cardiac hypertrophy. These results indicate that G3bp1-mediated inhibition of miR-1 processing with growth stimulation results in decrease in mature miR-1 and, thereby, an increase of its targets, which play fundamental roles in the development of hypertrophy.

**Conclusion:**

G3bp1 posttranscriptionally regulates miRNA-1 processing in the heart, and G3bp1 mediated downregulation of mature miRNA-1 levels is required for the derepression of its targets and increase in gene expression during cardiac hypertrophy.

## Introduction

Global increase in gene expression is the earliest and the most fundamental response to hypertrophic growth stimulus in heart [1–3]. Adaptations in gene expression can be regulated at the transcriptional level by change in the promoter activity [4–6] and chromatin remodeling [7,8], in addition, at the posttranscriptional level RNA binding proteins [9,10] and non-coding RNAs [11,12] have been identified as major players in regulating mRNA translation, stability and location.

MicroRNAs (miR) are small (~20–24 nucleotides), non-coding posttranscriptional regulators with the potential to regulate multiple genes by binding to their 3’ untranslated regions [13–16]. A single miR has been shown to target a group of functionally related genes, with more effective downstream effects as opposed to targeting of individual gene [17]. MiR-1 is a cardiac-enriched miR, and it accounts for ~40% of all miRs in the mouse heart [18] and 24% in human hearts [19]. Several studies have shown that miR-1 levels decrease with the development of cardiac hypertrophy [20–23], resulting in derepression of several hypertrophy related targets, some of which includes IGF1 [24], Mef2a, Calmodulin [25] and Twinfilin [19]. Recently, we identified and reported two targets of miR-1 in heart, Gtf2b and Cdk9, both essential components of the transcriptional machinery and hence required for transcription of all genes. We showed that downregulation of miR-1 is necessary for derepression of these targets and hence increase in transcription of hypertrophy-associated genes, however, it is not sufficient for the development of cardiac hypertrophy [26].

GTPase–activating protein (SH3 domain) binding protein 1 (G3bp1) was first identified as rasGAP binding protein [27], [28] and later shown to function as an endoribonuclease that selectively target genes by binding to its consensus sequence [29]. Expressed ubiquitously, G3bp1 function is regulated by rasGAP dependent phosphorylation at serine149. Hyperphosphorylation of G3bp1 is required for its endoribonuclease activity, while dephosphorylation favors the assembly of cytoplasmic protein-RNA aggregates called stress granules [30]. G3bp1 has also been shown to regulate the expression of Cdk7 in cardiomyocytes, by stabilizing Cdk7 mRNA [31].

Here we report that decrease in mature miR-1 in hypertrophied hearts is a result of a posttranscriptional regulation of miR processing, and not due to decrease in its transcription. We show that G3bp1 specifically targets pre-miR-1-2 transcript resulting in decrease in mature miR-1 levels. We have also identified eukaryotic initiation factor 4E (Eif4e) as a novel target of miR-1 in cardiomyocytes undergoing hypertrophy. Eif4e in upregulated in cardiomyocytes when subjected to hypertrophic stimuli [32] and phosphorylation of Eif4e has been shown necessary for cap-dependent translation [33,34], further, it was also shown necessary for cap-independent translation [35]. In this study we show that G3bp1 mediates downregulation of miR-1, which is necessary for the derepression of its targets that includes Eif4e and is required for the increase in protein synthesis during cardiac hypertrophy.

## Materials and Methods

### Animals

The work was done in accordance with the US National Institutes of Health Guidelines for the care and Use of Laboratory Animals (No 85–23). All animal protocols were approved by the Institutional Animal Care and Use Committee at Rutgers, the State University of New Jersey. C57Bl/6 mice were purchased from The Jackson Laboratory and were housed in Animal facility at Rutgers, The State University of New Jersey located in Newark NJ, as per standard procedures/protocols. The animals were fed autoclaved regular chow (5010) and receive Reverse Osmosis (RO) water.

### Transverse Aortic Constriction

Twelve-week-old male 92C57Bl/6 mice were anesthetized with a mixture of ketamine (65 mg/Kg), xylazine (13 mg/Kg) and acepromazine (2mg/Kg) via intraperitoneal injection. The adequacy of the anesthetic was confirmed by the loss of tongue retraction reflex. The transverse thoracic aorta between the innominate artery and the left common carotid artery was dissected free, and a 7–0 braided polyester suture was tied around the aorta against a 28-gause needle with the aid of an operating microscope. The needle was removed, the chest closed, and the mice were extubated and allowed to recover in a Thermocare unit (temperature 88°F or 31°C; humidity 30–50%; oxygen 1-2ml/min, low flow range). Postoperative buprenorphine (0.01–0.05 mg/kg) was administered subcutaneously every 12hrs, as needed. The sham operations involved the same procedure, except aorta was not constricted. Post surgery the animals were housed in Rutgers animal facility for the required time period. Animals were anaesthetized using ketamine (65 mg/Kg), xylazine (13 mg/Kg) and acepromazine (2mg/Kg) via intraperitoneal injection before hearts were isolated.

### ChIP-Seq of Sham and TAC-induced Hypertrophy Hearts and data analysis

As previously described in detail [26,36], sequencing data and analysis uploaded on NCBI Geo datasets (GSE50637 and GSE56813).

### Culturing Cardiomyocytes and Adenovirus infections

Cardiomyocytes were prepared as previously described [37]. Briefly, hearts were isolated from 1–2 day old Sprague-Dawley rats. After dissociation the cells are subjected to Percoll gradient centrifugation followed by differential pre-plating to enrich for cardiomyocytes and deplete non-myocytes. Myocytes are plated in DMEM-F12 with 10% fetal bovine serum (FBS) without antibiotics. Twenty-four hours after plating, the medium is changed and the cells are infected with recombinant adenoviruses at a multiplicity of infection (moi) of 10–20 particles / cell or as indicated.

### Construction of Adenoviruses

Recombinant adenoviruses were constructed, propagated and titered, as previously described by Dr. Frank Graham [38]. Briefly, pBHGloxΔE1,3Cre (Microbix), including the ΔE1 adenoviral genome, is co-transfected with the pDC shuttle vector containing the gene of interest, into 293 cells using Lipofectamine (Invitrogen). Through homologous recombination, the test genes integrate into the E1-deleted adenoviral genome. The viruses were propagated on 293 cells and purified using CsCl_2_ banding followed by dialysis against 20 mM Tris buffered saline with 2% glycerol. Titering is performed on 293 cells overlaid with Dulbeco’s Modified Eagle’s Medium (DMEM) plus 5% equine serum and 0.5% agarose.

### DNA constructs cloned into adenoviruses

MiR-1: the stem-loop of miR-1-2 was cloned into recombinant adenovirus under control of CMV promoter. MiR-1 eraser (anti-miR-1): two antisense repeats of the mature miR-1 sequence was synthesized as a double stranded oligonucleotide and cloned into recombinant adenovirus under the control of a U6 promoter. MiR-SC (scrambled control): the stem-loop expressing a scrambled sequence (GAACCGAGCCCACCAGCGAGC) was cloned into recombinant adenovirus under CMV and used as control for all adenoviral microRNA constructs. G3BP1: human cDNA was purchased from origene (SC127200) and cloned into recombinant adenovirus under CMV promoter. shRNA against G3bp1 (siG3bp1): hairpin forming oligonucleotides corresponding to bases 281–300 of mouse rasGAP SH3-binding protein (accession number NM_013716) and their antisense with Apa1- and HindIII- compatible overhangs were synthesized, annealed and subcloned distal to the U6 promoter.

### Northern Blots

Northern blots were performed as previously described [22]. Briefly, Total RNA (25–40μg), extracted using TRIzol reagent according to the protocol of the manufacturer (Invitrogen) was separated on 1% agarose gel with 3% formaldehyde and 10% 10X MOPS. The RNA was transferred onto uncharged nylon membrane and UV crosslinked. The membrane was prehybridized at 42°C for 2hrs with 1mL/cm^2^ MiracleHyp Hybridization solution (Agilent technologies). Radiolabelled (^32^P) DNA probes were used for hybridization (1x10^5^/cm^2^). The blot was hybridized overnight and washed with 2x SSC with 0.1% SDS and exposed to X-ray film for 24hrs at -80°C. The blots were stripped using 0.5% SDS for 1hr at 60°C and reprobed after prehybridization.

### Quantitative Polymerase Chain Reaction (qPCR)

Total RNA was reversed transcribed to cDNA using High Capacity cDNA Reverse Transcription Kit (applied Biosciences) as per manufacturers protocol. Quantitative PCR was performed using TaqMan gene expression assays (primer/probe sets) on Applied Biosystems 7500 Real-Time PCR system for the following genes; 18S (Mm03928990_g1), mmu-miR-1b (Mm03308741_pri), rno-miR-1 (Rn03465875_pri), pre-miR-1-2 (AILJJLS), mmu-miR-1-1 (Mm03306163_pri), has-miR-1 (RT: 2222), has-miR-21 (RT:000397), rno-miR-1 (TM:002064), U6 snRNA (TM: 001973), G3bp1 (Mm00785370_s1). Pre-miR-1-1 ordered from Integrated DNA technologies (set-1 assay, 57744637), rno-pre-miR-1-2 ordered from Integrated DNA technologies (5’primer: TGCCTACTCAGAGCACATAC; 3’primer: CACACTTCTTTACATTCCAT; probe: /56-FAM/TGTACCCAT/ZEN/ATGAACATAGAATGCT/3IABkFQ/).

### Cellular Fractionation and Western Blotting

Cells lysate were fractionated into cellular compartments using a Subcellular ProteoExtract Kit (Calbiochem), according to the manufacturer’s protocol. The protein was analyzed on a 4% to 20% gradient SDS-PAGE (Criterion gels, Bio-Rad) and transferred to nitrocellulose membrane for western blotting. Anti-G3bp1 (Millipore), Anti-Myosin (slow, skeletal) (Sigma), anti-phosphoAkt (Ser473) and anti-Akt (Cell Signaling), anti-Gapdh (Millipore), anti-Cdk9 (Santa Cruz Biotech), anti-Gtf2b (Millipore), anti-Ankrd1 (Santa Cruz Biotech), anti-Vdac1 (Genscript), anti-H2b (Upstate), anti-Eif4e (Genscript) and anti-Actin (Santa Cruz Biotech)

### Immunocytochemistry

Myocytes were fixed in 3% paraformaldehyde plus 0.3% triton X-100 in PBS at 25° for 20 minutes. They were the incubated with primary antibody in Tris-buffered saline with 1% BSA. After an overnight incubation, they were washed and the secondary antibody was added to the cells in TBS-1%BSA for additional 30minutes. The cells were washed, slides mounted using Prolong Gold Antifade with 4’,6-diamidino-2-phenylindole (DAPI) (Molecular Probes). Microscopy was performed on the fixed slides using Nikon Eclipse TS100 inverted microscope attached with CoolSNAP EZ CCD Camera (Photometrics) connected with NIS elements software. All images were taken using Nikon Plan Apo 60X A/1.40 Oil lens. Image J [39] was used for Eif4e particle analysis and cell area measurements.

### Statistics

Calculation of significance between 2 groups was performed using an unpaired, 2-tailed Student *t* test (excel software). All experiments were repeated three times, unless indicated and presented as average ± SEM *p*<0.05 was considered significant.

## Results

### MiR-1 expression is posttranscriptionally regulated during cardiac hypertrophy

**Fig 1:**
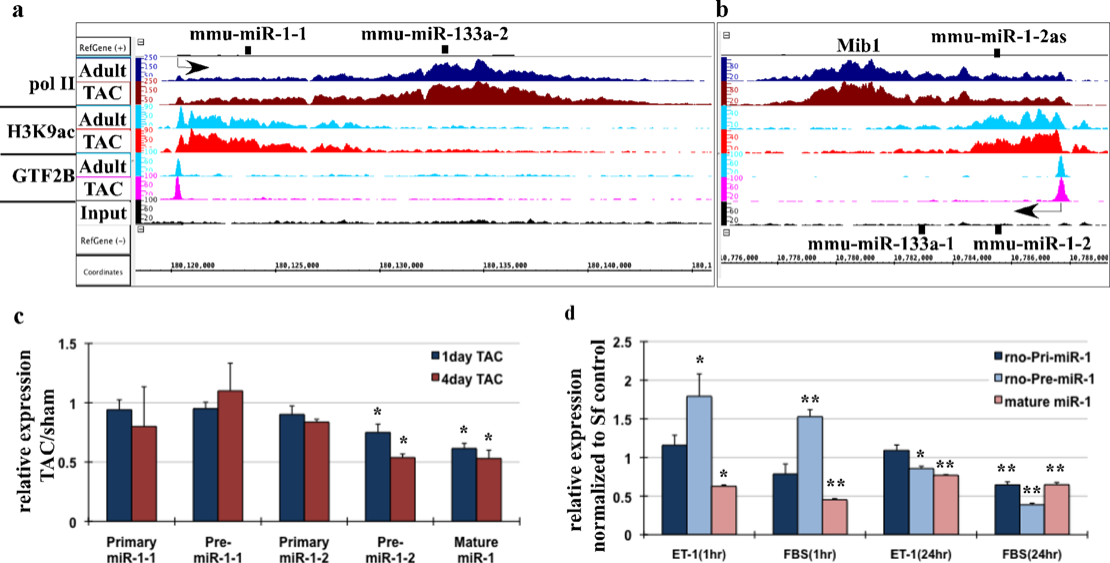
MiR-1 expression is posttranscriptionally regulated during cardiac hypertrophy. Pool of three sham/adult and TAC operated mouse hearts were used for anti-RNA pol II, anti-H3K9ac and anti-Gtf2b ChIP-Seq. Binary analysis files (BAR) from the ChIP-Seq data was viewed in Affymetrix’s Integrated Genome Browser (IGB), which shows the fragment densities of RNA pol II, H3K9ac and Gtf2b (*y-*axis) aligned in 32–50 nucleotide bins along the chromosomal coordinates (*x*-axis) for miR-1-133 clusters. The arrow indicates the transcription start site and the direction of transcription. (a) IGB images of miR-1-1 and miR-133a-2 transcript with RNA polII, H3K3ac and Gtf2b densities across intergenic regions of chromosome 2. (b) IGB images of miR-1-2 and miR-133a-1 cluster with RNA polII, H3K3ac and Gtf2b densities within the Mindbomb 1 (Mib1) gene located in chromosome 18. (c) Total mRNA extracted from mouse hearts from sham or TAC operated hearts for 1day or four days were used for qPCR analysis of primary, pre-miR-1-1, pre-miR-1-2 and mature miR-1 levels. The results were normalized to 18S (primary and pre- transcript) or U6 (mature) and shown as ratio of TAC/sham. Error bars represents standard error of mean (SEM) and * is p< 0.05, n = 3. (d) Neonatal myocytes cultured in growth-inhibited (serum free) conditions were stimulated with 100nM endothelin-1 or 10%FBS for 1hr or 24hrs, as indicated. Total RNA extracted was used for qPCR analysis of primary, pre- and mature miR-1. Error bars represents SEM, and * is p<0.05 and ** is p< 0.005. N = 3.

Others and we have shown that miR-1 levels are lower in the developing heart compared to the adult [22,40] and decreases when the adult heart undergoes hypertrophy in rodents and humans [20–22]. Paradoxically our RNA polII- and H3K9Ac-chromatin immunoprecipitation data [26,36] of the cardiac genome in mouse during pressure-induced cardiac growth compared to the sham hearts shows a minimal increase vs. decrease in the RNA pol II distribution across the miR-1-miR-133 clusters expressed from two chromosomal locations of the mouse genome, as well as, an equivalent increase in H3K9 acetylation and Gtf2b recruitment at the transcription start sites of these clusters. These results suggested that downregulation of mature miR-1 during cardiac hypertrophy could be due to regulatory posttranscriptional processing of primary or pre miR-1 transcript (Fig 1A and 1B). We quantified the primary, pre and mature miR-1 transcripts in mouse hearts subjected to sham or TAC operations for increasing time periods of 1day (no increase in heart weight/tibia length) or 4days (~50% increase in heart weight/tibia length) by quantitative PCR analysis of total RNA isolated and normalized to 18S (primary and pre) or U6 (mature). The results showed no significant change in the primary miR-1-1 and primary miR-1-2 transcripts in hypertrophied vs. sham hearts (Fig 1C). Interestingly, while pre-miR-1-1 did not change, pre-miR-1-2 transcript was decreased by 26% and 40% in both 1day and 4day TAC hearts, respectively compared to sham hearts. Similarly, mature miR-1 levels were 38% and 47% less in 1day and 4day TAC hearts vs. sham hearts. These results indicate that change in mature miR-1 levels in mouse hearts during cardiac hypertrophy is a result of posttranscriptional regulation of pre-miR-1 and not at transcriptional level. Similar regulation of miR-1 transcripts was seen in isolated neonatal rat ventricular cardiomyocytes stimulated with Endothelin-1 (ET-1) or 10% Fetal Bovine Serum (FBS) for 1hr or 24hrs. Interestingly, 1hr treatment showed no significant change in primary miR-1 transcript, with significant accumulation of pre-miR-1 and downregulation of mature miR-1 level. On the other hand, 24hrs treatment with ET-1 showed decrease in pre-miR-1 and mature miR-1, while FBS resulted in decrease in primary, pre and mature transcripts (Fig 1D). These results suggest that rapid decrease in mature miR-1 levels seen with induction of growth in cardiomyocytes is result of posttranscriptional regulation of pre-miR-1. Adenovirus mediated expression of exogenous miR-1 showed accumulation of pre-miR-1 in dose dependent manner, suggesting regulatory step before maturation (Figure a in S1 File). These results suggest that miR-1 levels are tightly controlled in cardiomyocytes, and are maintained by posttranscriptionally regulating miR-1 processing and maturation.

### MiR-1 stem-loop harbors the consensus sequence for G3bp1, an endoribonuclease with sequence-specific RNA binding activity

**Fig 2:**
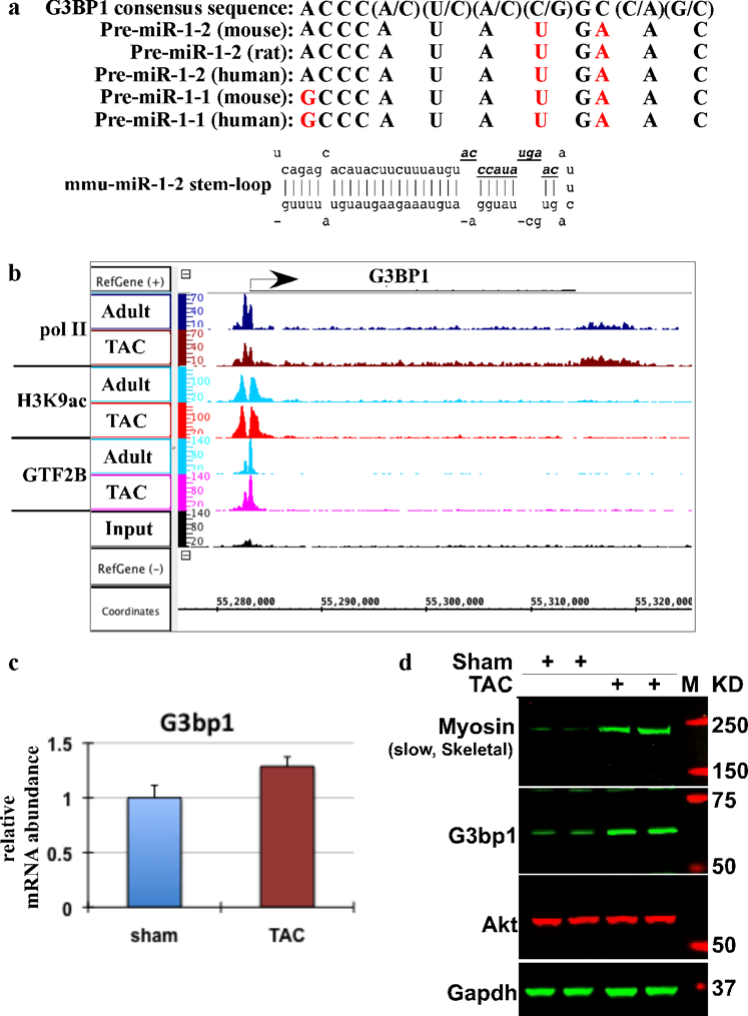
G3bp1 expression is increased during cardiac hypertrophy. (a) G3bp1 consensus sequence as identified by previous report aligned to the stem loop sequence of miR-1-1 and miR-1-2 of rat, mouse and human. (b) IGB image of RNA polII, H3K9ac and Gtf2b densities across G3bp1 gene in mouse chromosome 11. The *y*-axis represents the fragment density, while the *x*-axis chromosomal coordinates. (c) Total RNA from sham or TAC operated hearts (4days) was used to measure G3bp1 mRNA expression by qPCR. The graph represents the relative mRNA abundance, error bars represents SEM, n = 4. (d) Western blot analysis was performed on protein lysate extracted for the same hearts as in b. Blots were probed for the indicated genes. Myosin (slow, skeletal) that recognizes βMHC is shown as positive control for TAC, Akt as specificity and Gapdh as loading control. n = 4.

Our results show a rapid decrease in mature miR-1 levels immediately post TAC [22], (Fig 1), which suggest a posttranscriptional regulation of miR-1 in these hearts with induction of cardiac growth. Thus, we searched for potential posttranscriptional regulators that might be playing a role in miR-1 processing. While scanning the stem-loop sequence for target sites for RNA binding proteins with known consensus sequences, we discovered G3bp1, as a potential regulator of miR-1 processing. Studies have identified a consensus sequence for G3bp1, binding to which results in site directed cleavage of the target mRNA [29]. We identified this consensus sequence in pre-miR-1 transcript, and to our surprise, the sequence (with *two* nucleotide mismatch) was found in the stem-loop of miR-1-2. Aligning the loop sequences of rodent and human miR-1-1 and miR-1-2 confirmed conservation across species (Fig 2A). Interestingly, miR-1-1 stem-loop showed an additional mismatch in first nucleotide where G replaced an A. G3bp1 has been shown to cleave at CA dinucleotides [29]. Thus, we presumed that these differences might be the reason for differential regulation of pre-miR-1-1 and pre-miR-1-2 stem-loop by G3bp1, resulting in controlled decrease in mature miR-1 levels with induction of cardiac hypertrophy. Next, we examined the expression of G3bp1 in mouse hearts subjected to sham or TAC surgery for four days, as in Fig 1. Our RNA polII- and H3K9Ac-ChIP-Seq data showed that at the transcriptional level, G3bp1 is increased incrementally by promoter clearance of paused RNA pol II (Fig 2B), which results in incremental increase in G3bp1 transcript, (Fig 2C) and a ~3 fold increase in G3bp1 protein (Fig 2D). These results suggest that increase in G3bp1 with hypertrophic stress might be involved in the subsequent downregulation of mature miR-1, derepression of its hypertrophy related targets and induction of cardiac hypertrophy.

### G3bp1 regulates miR-1 processing in cardiomyocytes

**Fig 3:**
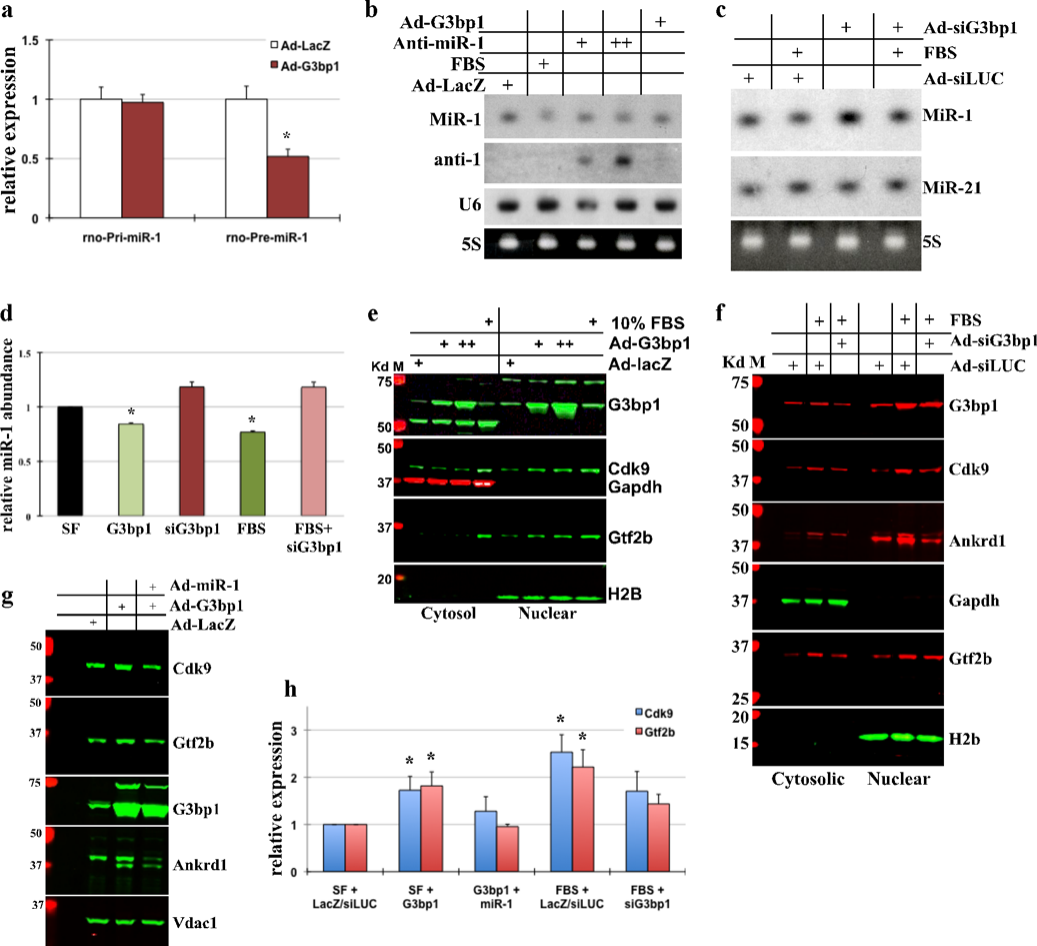
G3bp1 regulates miR-1 processing in cardiomyocytes. (a) Neonatal myocytes were treated with Ad-LacZ or Ad-G3bp1 for 24hrs; total RNA extracted was used for qPCR analysis for rno-primary and rno-pre-miR-1. The results were normalized to 18S, averaged and plotted. Error bars represents SEM, and * is p<0.05, n = 3. (b) Neonatal myocytes cultured in serum free conditions were treated with Ad-LacZ, Ad-G3bp1, Ad-anti-miR-1 (two doses, as indicated) or 10% FBS for 24hrs before extracting total RNA for Northern Blots. Blots were probed for miR-1, anti-miR-1 sequence or U6. 5S is shown as loading control. (c) Myocytes were cultured and treated with adenovirus expressing shRNA against G3bp1 (Ad-siG3bp), Ad-siLUC (control virus) or 10% FBS, as indicated. Northern blots were performed and probed for miR-1 and miR-21 (specificity control), 5S is shown as loading control. (d) Northern blots from 3a and b, and two independent blots were scanned, quantified, averaged and plotted. The error bars represents SEM and * p<0.05 vs. SF control. (e) Western blot analysis of the cytoplasmic and nuclear fractions of protein lysate was performed on myocytes for the indicated genes on myocytes treated with Ad-lacZ (control virus), Ad-G3bp1 or 10% FBS, as indicated for 24hrs. (f) Western blot analysis of cytoplasmic and nuclear fractions of protein lysate was performed on myocytes treated with Ad-siLUC (control virus), Ad-siG3BP1 or 10% FBS, as indicated. (g) Total protein lysate from cultured myocytes treated with Ad-lacZ (control virus), Ad-G3bp1 or Ad-miR-1, as indicated were separated by SDS-PAGE and protein expression of selected genes analyzed, n = 2. (h) Western blots were quantified, averaged and plotted. Error bars represents SEM and * p<0.05 vs. respective control. All experiments were performed in triplicates, unless indicated otherwise and representative blots have been shown.

To determine if G3bp1 regulates miR-1 processing, we expressed exogenous G3bp1 in neonatal myocytes and measured primary, pre-miR-1 and mature miR-1 levels. QPCR and Northern blot analysis showed no change in the primary miR-1 transcript, ~49% decrease in pre-miR-1 levels and ~25–30% decrease in the mature levels of miR-1 in myocytes treated with Ad-G3BP1, or with growth stimulation, compared to myocytes treated with Ad-LacZ (Fig 3A–3D and Figure b in S1 File). As expected, anti-miR-1 treatments showed decrease in miR-1 levels (Fig 3B). To further confirm these results, we tested if silencing G3bp1 with growth stimulation would result in the restored miR-1 processing and maintained mature miR-1 levels in cardiomyocytes. For that purpose, neonatal myocytes were infected with adenovirus expressing shRNA against G3bp1 in presence or absence of 10% FBS for 24hrs. The results showed that G3bp1 silencing increased mature miR-1 levels in absence of growth, and restored the serum-induced downregulation of miR-1 close to endogenous levels (Fig 3C and 3D). MiR-21 and U6 are shown for specificity, while 5S RNA as loading controls for Northern blots.

To examine if the change in G3bp1-mediated miR-1 levels is functionally relevant, we measured protein expression levels of validated downstream targets of miR-1, Gtf2b and Cdk9 [26] in cardiomyocytes cultured and treated as described above. Exogenous expression of G3bp1 in quiescent myocytes was sufficient to increase the expression of both Gtf2b and Cdk9, similar to growth stimulation by 10% FBS (Fig 3E and 3H, Figure c in S1 File). Conversely, shRNA mediated knockdown of G3bp1 partially restricted serum and growth factor induced upregulation of both Gtf2b and Cdk9 when stimulated with 10%FBS or ET-1 (Fig 3F and 3H, Figure d in S1 File). Gapdh and H2B are shown as specificity controls of cytosolic and nuclear fractions. These results suggests that G3bp1 by regulating miR-1 processing controls mature miR-1 levels and thereby, its downstream targets during cardiac hypertrophy development. To further examine that G3bp1-mediated changes in Gtf2b and Cdk9 protein expression were due to its role in regulating miR-1 processing, we supplemented quiescent neonatal myocytes infected with Ad-G3bp1 with exogenous miR-1. Supplementing exogenous miR-1 reversed G3bp1-induced upregulation of Gtf2b, Cdk9 and Ankrd1 (hypertrophic marker), while Vdac1 expression remained unchanged with both treatments (Fig 3G and 3H). Thus, we conclude that G3bp1 posttranscriptionally regulates miR-1 levels and hence its hypertrophy related targets.

### Mature MiR-1 level is inversely proportional to the growth status of cardiomyocytes and targets key transcriptional and translational modulators

**Fig 4:**
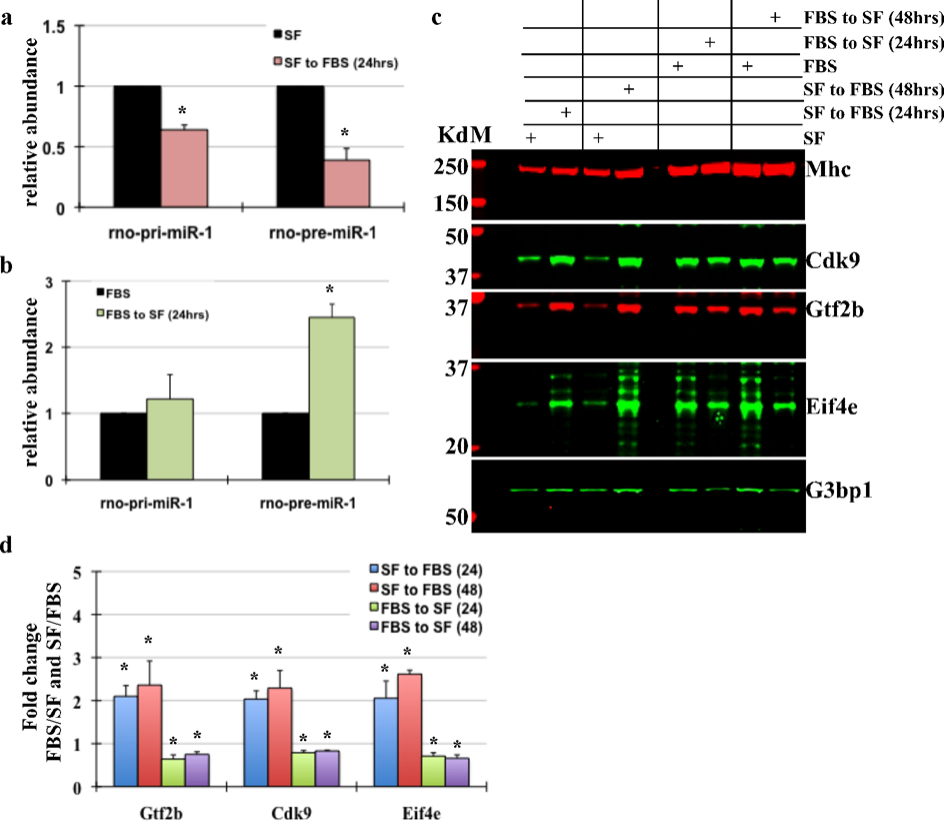
Mature MiR-1 level is inversely proportional to cardiomyocyte growth status. (a) Neonatal myocytes were cultured in growth-inhibited (serum-free, SF) medium for 24hrs before switching them to growth-stimulated medium (medium with 10% FBS) for an additional 24hrs. Total mRNA extracted was used for qPCR analysis of primary and pre-miR-1 expression. The graph represents relative abundance of primary and pre-miR-1 normalized to 18S, error bars represents SEM and * p<0.05 (n = 3). (b) Myocytes cultured in growth-stimulated medium (FBS) for 24hrs were switched to serum-free medium (SF) for additional 24hrs. Total mRNA extracted was used for qPCR analysis of primary and pre-miR-1 expression. The graph represents relative expression of primary and pre-miR-1 normalized to 18S, error bars represents SEM and * p<0.05 (n = 3). (c) Total protein was extracted from neonatal myocytes cultured similarly as in 4a and b with an additional 48hr time point, and separated by SDS-PAGE. Western blot analysis was performed for indicated genes. Myosin heavy Chain (Mhc) was used for loading control. (d) Western blot from 4c, and 2 other blots for the listed genes were quantified, averaged and plotted. The graph represents fold change normalized to Mhc signal and shown as ratio of FBS/SF or SF/FBS (n = 3). Error bars represents standard error of mean (SEM) and * is p<0.05 vs. respective controls. All experiments were repeated in triplicates and representative blots have been shown.

We recently showed that mature miR-1 levels are proportional to the growth status of cardiomyocytes, where switch to growth-stimulated condition (medium with 10% FBS) results in decrease in mature miR-1 and switch from growth stimulated to growth-inhibited medium (serum free medium, SF) results in a reciprocal increase in miR-1 levels [26]. To determine the status of primary and pre-miR-1 transcripts in cardiomyocytes cultured under similar conditions, we measured their abundance using qPCR. As seen in Fig 4A, growth-stimulated (FBS) condition results in a decrease in primary and pre-miR-1 transcript, whereas, a switch of hypertrophying myocytes to growth-inhibited medium showed a significant ~2.5fold increase in pre-miR-1 (Fig 4B), which may be due to decrease in G3bp1 endoribonuclease activity resulting in increase in pre-miR-1 and hence mature miR-1 levels. Thus, we conclude that miR-1 expression in cardiomyocytes inversely correlates with growth and is regulated posttranscriptionally at the pre-miR-1 processing step.

To validate that the change in miR-1 levels were functionally relevant we measured change in the protein expression of Gtf2b and Cdk9, and one predicted target Eif4e (predicted by both Targetscan and Pictar). While Gtf2b and Cdk9 are key regulators of transcriptional machinery, Eif4e is necessary for cap-dependent translation of mRNAs. Neonatal myocytes were cultured and treated similarly as above, with an additional 48hr time point, and western blot analysis was performed for the indicated genes. The results show that switch to growth-stimulated conditions (SF to FBS) that decreases mature miR-1 levels results in significant increase in Gtf2b, Cdk9 and Eif4e expression. On the other hand, removal of the growth stimulus (FBS to SF medium) that increases miR-1 reversed the expression levels of these selected targets (Fig 4C). Fig 4D shows the quantitative data of Gtf2b, Cdk9 and Eif4e normalized to myosin heavy chain (Mhc). We also examined total mRNA and total protein synthesis in neonatal myocytes subjected to growth stimulation in the absence or presence of exogenous miR-1 by measuring tritium radiolabelled Uridine (3H uridine) and Leucine (3H leucine), respectively. In addition, we treated quiescent myocytes with an adenovirus expressing anti-miR-1 sequence and measured total mRNA and protein synthesis. The results showed that miR-1 significantly reduced both mRNA and protein synthesis, while knockdown of miR-1 resulted in ~2.5fold increase Uridine incorporation, and minimal (~1.24 fold) increase protein synthesis (Figure a-c in S2 File).

### Eif4e is a direct target of miR-1

**Fig 5:**
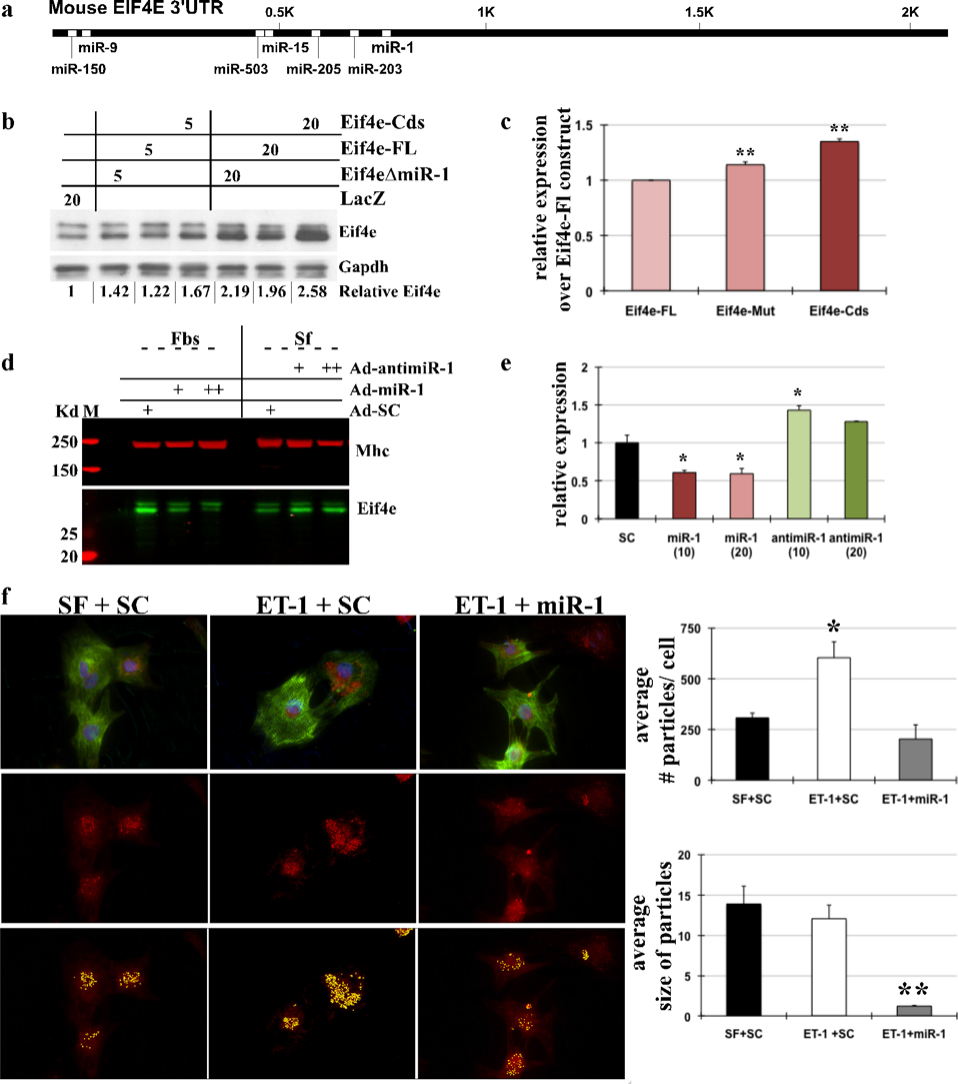
Eif4e is direct target of miR-1 in cardiomyocytes. (a) Targetscan and Pictar predicted miRs and sites on Eif4e 3’UTR that are broadly conserved between vertebrates. (b) Neonatal myocytes cultured in growth-inhibited medium (serum-free, SF) were infected with adenovirus constructs of Eif4e, as indicated, with multiplicity of infection (MOI) of 5 or 20 for 24hrs before protein extraction, followed by Western blotting for Eif4e and Gapdh. Relative Eif4e represents densitometry of two independent blots, averaged and normalized to Gapdh. (c) The graph represents relative increase in Eif4e protein expression levels with Eif4e-Mut and Eif4e-Cds constructs compared to Eif4e-Fl. Error bars represents SEM and ** is p<0.001, n = 3. (d) Neonatal myocytes were infected with Ad-SC, Ad-MiR-1 or Ad-anti-miR-1 in increasing doses in the presence or absence of 10% FBS, respectively, as indicated. Western blot analysis was performed on protein lysate for Eif4e and MHC. n = 3. (e) The graph represents change in Eif4e protein expression with Ad-miR1- or Ad-anti-miR-1 normalized to MHC. Error bars represents SEM and * is p<0.05, n = 3. (f) Immunocytochemistry was performed in myocytes plated in glass chamber slides and stimulated with ET-1 for 24hrs in the absence or presence of exogenous miR-1. Cells were stained for Eif4e, MHC and DAPI. Eif4e signal was quantified using Analyze particles tool from Image J. The graph represents average number of Eif4e stained particles/cell and average size of particles with the treatments. Error bars represent SEM, and * is p<0.05 and ** is p<0.005. Three independent experiments, four field each with more than 2cells used for quantification.

Both Targetscan [41–43] and Pictar [44,45] have predicted Eif4e as a target of miR-1, along with other miRs that are broadly conserved among vertebrates (Fig 5A). To validate Eif4e as direct target of miR-1 we made three adenoviral constructs of Eif4e cDNA that encompassed full length (Eif4e-FL), lacked the miR-1 target site (Eif4eΔmiR-1) or deleted 3’UTR (Eif4e-cds). These adenoviral vectors were used to infect neonatal myocytes cultured in growth-inhibited conditions that promote higher miR-1 levels (Figure a in S1 File). These myocytes were not supplemented with exogenous miR-1 and were infected with low (5 MOI) or high (20 MOI) doses of these constructs and expression of Eif4e measured. The data showed that constructs lacking the miR-1 target site or the 3’UTR had higher expression than the construct expressing the full length (FL) (Fig 5B and 5C). As expected, expression of Eif4e-cds was higher than Eif4eΔmiR-1, since there were no inhibitory effects of other predicted miRs like miR-15 or miR-150. While the lower dose showed ~20% and ~35% more expression of Eif4eΔmiR-1 and Eif4e-cds, respectively, over Eif4e-FL, increasing concentrations of constructs showed reduced difference in expression levels which may be due to the ‘sponging effect’.

To further verify that miR-1 regulates the expression of Eif4e in cardiomyocytes during growth, we supplemented neonatal myocytes with exogenous miR-1 in increasing doses. The results showed that miR-1 inhibited expression of endogenous Eif4e protein levels (Fig 5D and 5E). In concordance, inhibition of miR-1 expression by an anti-miR-1 sequence in quiescent neonatal myocytes resulted in increase in Eif4e in dose dependent manner. Mhc is shown as loading control (Fig 5D and 5E). Next, we performed immunocytochemistry in neonatal myocytes stimulated with growth factor, Endothelin 1 (ET-1) in the absence or presence of exogenous miR-1. Cardiomyocytes stained with Eif4e, Myosin heavy chain (MF20) and DAPI showed that addition of miR-1 resulted in inhibition of ET-1 induced increase in cell size and decrease in the Eif4e expression (Fig 5F and Figure f in S1 File). We measured Eif4e expression changes with these treatments using ImageJ, which showed an increase in average number of Eif4e staining seen as particles per cardiomyocyte with ET-1 stimulation that was decreased to basal levels in presence of exogenous miR-1. Interestingly, the results revealed significant decrease in average size of particles in the presence of exogenous miR-1, which was not seen in quiescent or stimulated cardiomyocytes alone (Fig 5F). Thus, these data suggest that miR-1 directly targets Eif4e in cardiomyocytes and may regulate cap-dependent translation.

**Fig 6:**
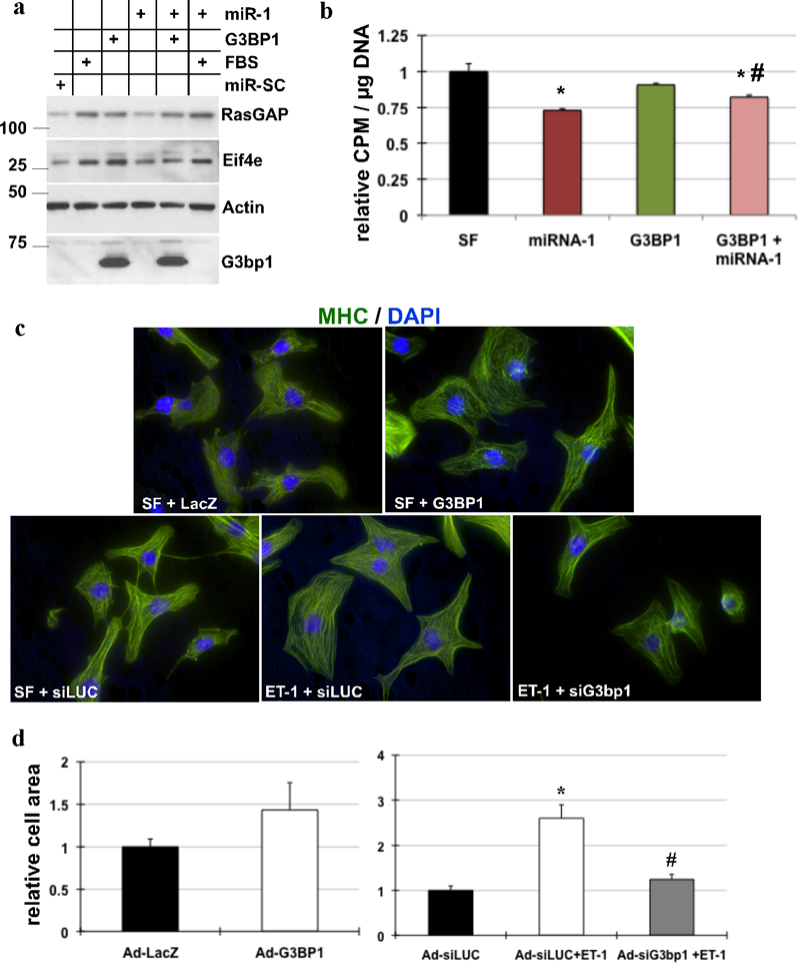
Inhibition of G3bp1 restricts ET-1 induced cardiac hypertrophy. (a) Neonatal myocytes were infected with adenoviruses expressing miR-SC (control virus), G3BP1, miR-1 or 10% FBS, as indicated. Total protein lysate was extracted and analyzed by western blotting for Eif4e, rasGAP, Actin and G3bp1 (n = 2). (b) Neonatal myocytes cultured were treated with adenovirus expressing miR-1, G3BP1 or control virus, as indicated. After 24hrs the cells were supplemented with [H^3^]-leucine to the medium. After additional 24hrs, protein and DNA was extracted and counts per minute (CPM) measured, normalized to DNA, averaged and plotted. Graph represents relative CPM/μg of DNA Error Bars represents SEM, * represents p<0.05 vs. control. # represents p<0.05 G3bp1+miR-1 vs. miR-1 (n = 3). (c) Immunocytochemistry was performed on myocytes plated in glass chamber slides and treated with Ad-LacZ or Ad-G3BP1, as indicated. Myocytes were stimulated with ET-1 in the presence or absence of Ad-siG3bp1 or Ad-siLUC. The cells were fixed and stained for phalloidin and DAPI. N = 3. d. The graph represents cell area quantified using Image J, error bars represents SEM and * is p<0.05 vs. Ad-siLUC, and # is p<0.05 vs. ET-1+Ad-siLUC, n = 3 independent experiments.

To examine the effects of G3bp1 on Eif4e protein expression and if restoring miR-1 levels by supplementing exogenous miR-1 would rescue the effects, we treated neonatal myocytes cultured under serum free conditions with ad-G3bp1 or 10% FBS in the absence or presence of ad-miR-1. The results showed that growth stimulation and G3bp1 resulted in increase Eif4e expression; however, the presence of exogenous miR-1 restricted this increase in both these treatments (Fig 6A). Interestingly, supplementing exogenous G3bp1 to myocytes restored miR-1 induced inhibition of total protein synthesis, however, G3bp1 alone was not sufficient to increase protein synthesis (Fig 6B). These results suggest that G3bp1 mediated downregulation of miR-1 is required for increase in Eif4e expression in cardiomyocytes, which is necessary to accommodate the increase in translation of mRNA transcripts (Figure d in S2 File).

### Inhibition of G3BP1 abrogates growth factor induced cardiac hypertrophy

We have shown that exogenous miR-1 restricts ET-1 induced hypertrophy in cardiomyocytes [22], [26], (Fig 5F). Since inhibition of G3bp1 results in increase in mature miR-1 levels, we tested if inhibiting endogenous G3bp1 in cultured neonatal myocytes would also restrict ET-1 induced increase in cell size. Immunocytochemistry in cultured neonatal myocytes treated with ad-siG3bp1 in the presence of ET-1 confirmed that G3bp1 was required for development of cardiac hypertrophy (Fig 6C). Interestingly, overexpression of G3bp1 was not sufficient for the development of cardiac hypertrophy (Fig 6C). These results are in concordance of with our previous study with miR-1, where downregulation of miR-1 is not sufficient for hypertrophy development in vivo [26], but necessary for cardiac hypertrophy. Thus, these results signify the essential function of G3bp1 in regulating miR-1 processing, and hence its role in the development of cardiac hypertrophy.

## Discussion

In this study we show that downregulation of miR-1 with induction of cardiac hypertrophy is due to posttranscriptional regulation of pre-miR-1 by an endoribonuclease G3BP1 and not due to change in transcription of the primary transcript.

### MiR-1 expression and regulation

Transcription of majority of the miRs is RNA pol II dependent and follows similar processing as most mRNAs, which includes capping, splicing and polyadenylation [46]. The coding regions of miRs can be intergenic or within genes, and can be transcribed independently or with the host gene as an individual or cluster of several miRs, which is later spliced and processed for the expression of mature miR [47]. MiR-1 is expressed as a co-transcript with miR-133 from two chromosomal locations (Fig 1A and 1B), well conserved in mouse and humans [40]. Although, both these miRs have been reported as cardiac-enriched, mature miR-1 levels significantly exceeds those of miR-133 and accounts for ~40% of all cardiac miRs in mice, suggesting miR-1-specific processing of the primary transcript [18]. Similar specific regulation of miR clusters have been reported, like the differential expression of miRs of the miR-17~92 cluster, where hnRNP A1, an RNA binding protein has been shown to specifically facilitate processing of only miR-18a by binding to the loop sequence in primary transcript and resulting in conformational change that favors Drosha processing, without affecting the expression of miR-17 or miR-19 [48,49]. It has been estimated that 14% of pri-miRs have conserved loop sequence, which includes miR-1-2 and suggests posttranscriptional regulation of its biogenesis [49]. Several previous studies have implicated RNA binding proteins as regulators of miR-1 biogenesis that play a critical role in maintaining and regulating mature miR-1 levels. KH-type splicing regulatory protein (KSRP), a nuclear and cytoplasmic nucleic acid binding protein that specifically targets AU rich regions and regulate mRNA decay [50] has been shown to promote miR-1 biogenesis in C2C12 cells. Pri-miR-1 is part of bigger group that includes several miRs that require KSRP binding at the specific sequences (G repeats) in the terminal-loop, this facilitates Drosha and Dicer mediated miR processing, without binding to single or double stranded mature miR [51]. An independent study however reported that KSRP also associates with mature miR-1 sequence in C2C12 cells [52]. A posttranscriptional regulation of miR-1 has also been reported during myotonic dysfunction induced cardiac defects. Muscleblind-Like Splicing Regulator 1 (MBNL1) has been shown to compete with Lin28 in H9C2 rat cardiomyocytes for specific G- and A- rich regions in the terminal-loop of pre-miR-1, thereby promoting or inhibiting pre-miR-1 processing, respectively [53]. On the other hand, transactive response DNA binding protein 43 (TDP-43) negatively regulates miR-1 activity in skeletal muscle without altering mature miR-1 abundance [52]. The modus operandi of these factors in regulating miR-1 involves binding to the terminal-loop sequence of the pre-miR-1, except for TDP-43 that prevents incorporation of mature miR-1 into the RISC complex. Here we show that the previously identified binding consensus sequence for G3bp1 also stretches across the terminal-loop of pre-miR-1, and an increase in the endogenous G3bp1 results in decrease in specifically pre-miR-1-2 abundance and hence decrease in mature miR-1 with hypertrophic stimulation in cardiomyocytes.

### G3bp1 mediated posttranscriptional regulation of RNA

G3bp1 belongs to the superfamily of heterogenous RNA binding protein (hnRNP) with two-riboncleoprotein motifs and RGG (arginine-glycine-glycine)–rich domain, but lacking the KH domain [27]. Further characterization has also identified nuclear transfer factor like 1 and acidic domain that harbors serine 149 residue, a phosphorylation of which regulates G3bp1 function and subcellular location [29,54,55]. G3bp1 has binding specificity for RNAs harboring consensus sequence ACCC(A/C)(U/C)(A/C)(C/G)G(C/A)A(G/C) within their 3’UTR, with the cleavage site between CA dinucletoides [29]. Apart from its function as an endoribonuclease, G3bp1 is also a core component of stress granules and several studies have reported G3bp1 as necessary and sufficient for nucleation of these granules in stressed cells. Studies in fibroblasts have shown that hyperphosphorylation of G3bp1 is required for endoribonuclease activity, while dephosphorylation favors assembly of stress granules [30,56,57]. G3bp1 has relatively high expression in the heart, and according to our data is increased during pressure-induced cardiac hypertrophy in mice. At the transcriptional level, our ChIP-Seq data shows that G3bp1 is regulated by release of promoter paused RNA pol II, which results in 0.2 fold increase in G3bp1 mRNA, as expected. This mode of gene regulation is widespread in the heart and mostly seen in essential/housekeeping genes during cardiac hypertrophy, oppose to de novo recruitment of RNA pol II to gene promoters observed in a small subset of specialized genes [26,36]. RNA immunoprecipitation with G3bp1 would be ideal to validate the direct binding of G3bp1 with pre-miR-1-2, however; we were unable to precipitate pre-miR-1-2 with G3bp1, which may be due to degradation of the transcript by G3bp1 under the tested conditions. In addition, it would be interesting to see if stress granules are assembled in cardiomyocytes during hypertrophy and if pre-miR-1 transcript/s are first recruited to stress granules by G3bp1, before selective sequence-dependent degradation of pre-miR-1-2.

General knockout of G3BP is embryonic lethal with fetal growth retardation and extensive neuronal death [58]. Microarray on mouse embryonic fibroblasts from knockout mice identified five genes (Gas5, Insulin-like growth factor II, secreted frizzled-related protein 2, delta-like homolog 1 and Lumican) with more than four fold upregulation compared to control in resting and serum stimulated cells, in addition these genes also contained G3bp consensus sequence [58]. Other known targets of G3bp1 include β-f1 ATPase mRNA [59] and peripheral myelin protein 22 [60] in HEK and MCF7 cells, respectively. In the heart, the only reports of G3bp1 is with respect to its expression [27,55] and its role in regulation of Cdk7 and Cdk9 during cardiomyocyte growth [31]. Here we show that G3bp1 mediated rapid downregulation of pre-miR-1-2 is essential for the derepression of miR-1 targets that includes Gtf2b, Cdk9 and Eif4e, all of which play an essential role in global gene expression. In agreement with our previous data showing that downregulation of miR-1 is not sufficient for induction of cardiac hypertrophy [26], overexpression of exogenous G3bp1 in cardiomyocytes did not result in increase cell size or increase in total protein synthesis. However, inhibition of endogenous G3bp1 that increases mature miR-1 levels restricts endothelin1 induced cardiomyocyte hypertrophy, as seen with miR-1 overexpression [22,26]. Therefore we conclude that G3bp1 plays an essential role in maintaining the levels of mature miR-1 in cardiomyocytes. A hypertrophic stimulus induces an increase in G3bp1, which mediates a selective decrease in pre-miR-1-2 and hence, decrease in mature miR-1 levels. Downregulation of mature miR-1 is necessary for derepression of keys transcriptional and translational targets that are required for compensatory increase in global gene expression, one of the hallmarks of cardiac hypertrophy.

## Supporting Information

S1 Checklist (PDF)

S1 File G3bp1 regulates mature miR-1 levels and hence downstream targets. Figure a. Neonatal myocytes cultured in serum free conditions were infected with increasing doses of Ad-miR-1 for 24hrs, total RNA was extracted and was used for Northern Blot analysis of miR-1 5S is shown as loading control. Figure b. QPCR for miR-1 was performed on total RNA extracted from neonatal myocytes treated with Ad-LacZ or Ad-G3bp1. The graph represents relative miR-1 levels normalized to U6. Error bars represents SEM, and * is p<0.05, n = 3. Figure c. Total protein lysate from neonatal myocytes treated with Ad-LacZ or Ad-G3bp1 was separated by SDS-PAGE and western blotting performed for indicated genes. Figure d. Neonatal myocytes were stimulated with 100nM ET-1 or 10% FBS for 1hr in the presence or absence of Ad-siG3bp1 or Ad-siLUC. Total protein lysate from the cells were separated by SDS-PAGE and western blotting performed for indicated genes. Figure e. QPCR was performed to validate the downregulation of G3bp1 with Ad-siG3bp1 in neonatal myocytes. The graph represents relative G3bp1 mRNA abundance. Error bars represents SEM and ** is p<0.00001, n = 3. Figure f. Immunocytochemistry on neonatal myocytes stimulated with ET-1 in presence of Ad-Sc or Ad-miR-1, and stained for Eif4e, Mhc and Dapi.
(TIFF)

S2 File MiR-1 regulates mRNA and total protein synthesis in cardiomyocytes. Figure a. Neonatal myocytes cultured under growth-inhibited conditions were infected with adenoviruses expressing miR-Sc, miR-1 or anti-miR-1 for 24hrs in the presence of [H^3^]-Uridine before extracting total RNA. mRNA was separated using Oligotex Direct mRNA kit from Qiagen. Counts per minute (CPM) was measured and normalized to CPM of total RNA, averaged and plotted. Error bars represents SEM and * is p<0.05, n = 3. Figure b and Figure c. Neonatal myocytes were infected with miR-Sc, miR-1 or anti-miR-1 as indicated, in the presence of [H^3^]-leucine. After 24hrs protein and DNA was extracted and [H^3^]-leucine incorporation measured by a scintillation counter. CPM was measured and normalized to DNA, averaged and plotted. Error bars represents SEM and * is p<0.05, n = 3. Figure d. Neonatal myocytes cultured in growth-inhibited conditions were treated with siLUC or siEif4e. After 24hrs, cells were supplemented with [H^3^]-leucine and 10% fetal bovine serum for additional 24hrs. Protein and DNA was precipitated and extracted as per protocol, and incorporation of [H^3^]-leucine measured and plotted as described above. Error bars represents SEM and * is p<0.05 vs. their respective controls. # is p<0.01 siEif4e with FBS vs. siLUC with FBS. The graph also indicates the percent increase in protein synthesis after FBS treatment in cardiomyocytes after treatments with siLUC or siEif4e, as indicated.
(TIFF)

## References

[1] Chien KR, Knowlton KU, Zhu H, Chien S. Regulation of cardiac gene expression during myocardial growth and hypertrophy: molecular studies of an adaptive physiologic response. Faseb J. 1991;5(15):3037–46. 1835945 10.1096/fasebj.5.15.1835945

[2] Cutilletta AF. Changes in transcriptional activity during myocardial hypertrophy. Tex Rep Biol Med. 1979;39:95–109. 162250

[3] Meerson FZ, Javitz MP, Breger AM, Lerman MI. The mechanism of the heart's adaption to prolonged load and dynamics of RNA synthesis in the myocardium. Basic Res Cardiol. 1974;69(5):484–99. 4280903 10.1007/BF01906981

[4] Cutilletta AF. Regression of myocardial hypertrophy. II. RNA synthesis and RNA polymerase activity. J Mol Cell Cardiol. 1980;12(8):827–32. 6158574 10.1016/0022-2828(80)90083-8

[5] Cutilletta AF, Rudnik M, Zak R. Muscle and non-muscle cell RNA polymerase activity during the development of myocardial hypertrophy. J Mol Cell Cardiol. 1978;10(8):677–87. 151746 10.1016/0022-2828(78)90403-0

[6] Pikkarainen S, Tokola H, Kerkela R, Ruskoaho H. GATA transcription factors in the developing and adult heart. Cardiovasc Res. 2004;63(2):196–207. 15249177 10.1016/j.cardiores.2004.03.025

[7] Backs J, Olson EN. Control of cardiac growth by histone acetylation/deacetylation. Circ Res. 2006;98(1):15–24. 16397154 10.1161/01.RES.0000197782.21444.8f

[8] Mahmoud SA, Poizat C. Epigenetics and chromatin remodeling in adult cardiomyopathy. J Pathol. 2013;231(2):147–57. 10.1002/path.4234 23813473 PMC4285861

[9] Chang SH, Hla T. Post-transcriptional gene regulation by HuR and microRNAs in angiogenesis. Curr Opin Hematol. 2014;21(3):235–40. 10.1097/MOH.0000000000000040 24714527

[10] Giudice J, Cooper TA. RNA-binding proteins in heart development. Adv Exp Med Biol. 2014;825:389–429. 10.1007/978-1-4939-1221-6_11 25201112

[11] Ho JJ, Marsden PA. Competition and collaboration between RNA-binding proteins and microRNAs. Wiley Interdiscip Rev RNA. 2014;5(1):69–86. 10.1002/wrna.1197 24124109

[12] Yoon JH, Abdelmohsen K, Gorospe M. Posttranscriptional gene regulation by long noncoding RNA. J Mol Biol. 2013;425(19):3723–30. 10.1016/j.jmb.2012.11.024 23178169 PMC3594629

[13] Lau NC, Lim LP, Weinstein EG, Bartel DP. An abundant class of tiny RNAs with probable regulatory roles in Caenorhabditis elegans. Science. 2001;294(5543):858–62. 11679671 10.1126/science.1065062

[14] Lee RC, Ambros V. An extensive class of small RNAs in Caenorhabditis elegans. Science. 2001;294(5543):862–4. 11679672 10.1126/science.1065329

[15] Lee RC, Feinbaum RL, Ambros V. The C. elegans heterochronic gene lin-4 encodes small RNAs with antisense complementarity to lin-14. Cell. 1993;75(5):843–54. 8252621 10.1016/0092-8674(93)90529-y

[16] Sayed D, Abdellatif M. MicroRNAs in Development and Disease. Physiol Rev. 2011;91(3):827–87. 10.1152/physrev.00006.2010 21742789

[17] Liu Q, Fu H, Sun F, Zhang H, Tie Y, Zhu J, et al miR-16 family induces cell cycle arrest by regulating multiple cell cycle genes. Nucleic Acids Res. 2008;36(16):5391–404. 10.1093/nar/gkn522 18701644 PMC2532718

[18] Rao PK, Toyama Y, Chiang HR, Gupta S, Bauer M, Medvid R, et al Loss of cardiac microRNA-mediated regulation leads to dilated cardiomyopathy and heart failure. Circ Res. 2009;105(6):585–94. 10.1161/CIRCRESAHA.109.200451 19679836 PMC2828903

[19] Li Q, Song XW, Zou J, Wang GK, Kremneva E, Li XQ, et al Attenuation of microRNA-1 derepresses the cytoskeleton regulatory protein twinfilin-1 to provoke cardiac hypertrophy. J Cell Sci. 2010;123(Pt 14):2444–52. 10.1242/jcs.067165 20571053

[20] Care A, Catalucci D, Felicetti F, Bonci D, Addario A, Gallo P, et al MicroRNA-133 controls cardiac hypertrophy. Nat Med. 2007;13(5):613–8. 17468766 10.1038/nm1582

[21] Ikeda S, Kong SW, Lu J, Bisping E, Zhang H, Allen PD, et al Altered microRNA expression in human heart disease. Physiol Genomics. 2007;31(3):367–73. 17712037 10.1152/physiolgenomics.00144.2007

[22] Sayed D, Hong C, Chen IY, Lypowy J, Abdellatif M. MicroRNAs play an essential role in the development of cardiac hypertrophy. Circ Res. 2007;100(3):416–24. 17234972 10.1161/01.RES.0000257913.42552.23

[23] Tatsuguchi M, Seok HY, Callis TE, Thomson JM, Chen JF, Newman M, et al Expression of microRNAs is dynamically regulated during cardiomyocyte hypertrophy. J Mol Cell Cardiol. 2007;42(6):1137–41. 17498736 10.1016/j.yjmcc.2007.04.004 PMC1934409

[24] Elia L, Contu R, Quintavalle M, Varrone F, Chimenti C, Russo MA, et al Reciprocal regulation of microRNA-1 and insulin-like growth factor-1 signal transduction cascade in cardiac and skeletal muscle in physiological and pathological conditions. Circulation. 2009;120(23):2377–85. 10.1161/CIRCULATIONAHA.109.879429 19933931 PMC2825656

[25] Ikeda S, He A, Kong SW, Lu J, Bejar R, Bodyak N, et al MicroRNA-1 negatively regulates expression of the hypertrophy-associated calmodulin and Mef2a genes. Mol Cell Biol. 2009;29(8):2193–204. 10.1128/MCB.01222-08 19188439 PMC2663304

[26] Sayed D, Yang Z, He M, Pfleger JM, Abdellatif M. Acute targeting of general transcription factor IIB restricts cardiac hypertrophy via selective inhibition of gene transcription. Circ Heart Fail. 2015;8(1):138–48. 10.1161/CIRCHEARTFAILURE.114.001660 25398966 PMC4401077

[27] Parker F, Maurier F, Delumeau I, Duchesne M, Faucher D, Debussche L, et al A Ras-GTPase-activating protein SH3-domain-binding protein. Mol Cell Biol. 1996;16(6):2561–9. 8649363 10.1128/mcb.16.6.2561 PMC231246

[28] Kennedy D, Wood SA, Ramsdale T, Tam PP, Steiner KA, Mattick JS. Identification of a mouse orthologue of the human ras-GAP-SH3-domain binding protein and structural confirmation that these proteins contain an RNA recognition motif. Biomed Pept Proteins Nucleic Acids. 1996;2(3):93–9. 9575347

[29] Tourriere H, Gallouzi IE, Chebli K, Capony JP, Mouaikel J, van der Geer P, et al RasGAP-associated endoribonuclease G3Bp: selective RNA degradation and phosphorylation-dependent localization. Mol Cell Biol. 2001;21(22):7747–60. 11604510 10.1128/MCB.21.22.7747-7760.2001 PMC99945

[30] Tourriere H, Chebli K, Zekri L, Courselaud B, Blanchard JM, Bertrand E, et al The RasGAP-associated endoribonuclease G3BP assembles stress granules. J Cell Biol. 2003;160(6):823–31. 12642610 10.1083/jcb.200212128 PMC2173781

[31] Lypowy J, Chen IY, Abdellatif M. An alliance between Ras GTPase-activating protein, filamin C, and Ras GTPase-activating protein SH3 domain-binding protein regulates myocyte growth. J Biol Chem. 2005;280(27):25717–28. 15886195 10.1074/jbc.M414266200

[32] Makhlouf AA, McDermott PJ. Increased expression of eukaryotic initiation factor 4E during growth of neonatal rat cardiocytes in vitro. Am J Physiol. 1998;274(6 Pt 2):H2133–42. 9841540 10.1152/ajpheart.1998.274.6.H2133

[33] Sonenberg N, Gingras AC. The mRNA 5' cap-binding protein eIF4E and control of cell growth. Curr Opin Cell Biol. 1998;10(2):268–75. 9561852 10.1016/s0955-0674(98)80150-6

[34] Wada H, Ivester CT, Carabello BA, Cooper Gt, McDermott PJ. Translational initiation factor eIF-4E. A link between cardiac load and protein synthesis. J Biol Chem. 1996;271(14):8359–64. 8626533 10.1074/jbc.271.14.8359

[35] Scheper GC, Voorma HO, Thomas AA. Eukaryotic initiation factors-4E and -4F stimulate 5' cap-dependent as well as internal initiation of protein synthesis. J Biol Chem. 1992;267(11):7269–74. 1559971

[36] Sayed D, He M, Yang Z, Lin L, Abdellatif M. Transcriptional regulation patterns revealed by high resolution chromatin immunoprecipitation during cardiac hypertrophy. J Biol Chem. 2013;288(4):2546–58. 10.1074/jbc.M112.429449 23229551 PMC3554922

[37] Sayed D, Rane S, Lypowy J, He M, Chen IY, Vashistha H, et al MicroRNA-21 targets Sprouty2 and promotes cellular outgrowths. Mol Biol Cell. 2008;19(8):3272–82. 10.1091/mbc.E08-02-0159 18508928 PMC2488276

[38] Graham F. L. PL. Methods in Molecular Biology. The Humana Press Inc, Clifton, NJ 1991:109–28. 10.1385/0-89603-178-0:109 21416352

[39] Schneider CA, Rasband WS, Eliceiri KW. NIH Image to ImageJ: 25 years of image analysis. Nat Methods. 2012;9(7):671–5. 22930834 10.1038/nmeth.2089 PMC5554542

[40] Chen JF, Mandel EM, Thomson JM, Wu Q, Callis TE, Hammond SM, et al The role of microRNA-1 and microRNA-133 in skeletal muscle proliferation and differentiation. Nat Genet. 2006;38(2):228–33. 16380711 10.1038/ng1725 PMC2538576

[41] Friedman RC, Farh KK, Burge CB, Bartel DP. Most mammalian mRNAs are conserved targets of microRNAs. Genome Res. 2009;19(1):92–105. 10.1101/gr.082701.108 18955434 PMC2612969

[42] Grimson A, Farh KK, Johnston WK, Garrett-Engele P, Lim LP, Bartel DP. MicroRNA targeting specificity in mammals: determinants beyond seed pairing. Mol Cell. 2007;27(1):91–105. 17612493 10.1016/j.molcel.2007.06.017 PMC3800283

[43] Lewis BP, Burge CB, Bartel DP. Conserved seed pairing, often flanked by adenosines, indicates that thousands of human genes are microRNA targets. Cell. 2005;120(1):15–20. 15652477 10.1016/j.cell.2004.12.035

[44] Creighton CJ, Nagaraja AK, Hanash SM, Matzuk MM, Gunaratne PH. A bioinformatics tool for linking gene expression profiling results with public databases of microRNA target predictions. Rna. 2008;14(11):2290–6. 10.1261/rna.1188208 18812437 PMC2578856

[45] Krek A, Grun D, Poy MN, Wolf R, Rosenberg L, Epstein EJ, et al Combinatorial microRNA target predictions. Nat Genet. 2005;37(5):495–500. 15806104 10.1038/ng1536

[46] Lee Y, Kim M, Han J, Yeom KH, Lee S, Baek SH, et al MicroRNA genes are transcribed by RNA polymerase II. Embo J. 2004;23(20):4051–60. 15372072 10.1038/sj.emboj.7600385 PMC524334

[47] Kim VN. MicroRNA biogenesis: coordinated cropping and dicing. Nat Rev Mol Cell Biol. 2005;6(5):376–85. 15852042 10.1038/nrm1644

[48] Guil S, Caceres JF. The multifunctional RNA-binding protein hnRNP A1 is required for processing of miR-18a. Nat Struct Mol Biol. 2007;14(7):591–6. 17558416 10.1038/nsmb1250

[49] Michlewski G, Guil S, Semple CA, Caceres JF. Posttranscriptional regulation of miRNAs harboring conserved terminal loops. Mol Cell. 2008;32(3):383–93. 10.1016/j.molcel.2008.10.013 18995836 PMC2631628

[50] Briata P, Chen CY, Ramos A, Gherzi R. Functional and molecular insights into KSRP function in mRNA decay. Biochim Biophys Acta. 2013:22. 10.1016/j.bbagrm.2012.11.003 23178464

[51] Trabucchi M, Briata P, Garcia-Mayoral M, Haase AD, Filipowicz W, Ramos A, et al The RNA-binding protein KSRP promotes the biogenesis of a subset of microRNAs. Nature. 2009;459(7249):1010–4. 10.1038/nature08025 19458619 PMC2768332

[52] King IN, Yartseva V, Salas D, Kumar A, Heidersbach A, Ando DM, et al The RNA-binding protein TDP-43 selectively disrupts microRNA-1/206 incorporation into the RNA-induced silencing complex. J Biol Chem. 2014;289(20):14263–71. 10.1074/jbc.M114.561902 24719334 PMC4022891

[53] Rau F, Freyermuth F, Fugier C, Villemin JP, Fischer MC, Jost B, et al Misregulation of miR-1 processing is associated with heart defects in myotonic dystrophy. Nat Struct Mol Biol. 2011;18(7):840–5. 10.1038/nsmb.2067 21685920

[54] Gallouzi IE, Parker F, Chebli K, Maurier F, Labourier E, Barlat I, et al A novel phosphorylation-dependent RNase activity of GAP-SH3 binding protein: a potential link between signal transduction and RNA stability. Mol Cell Biol. 1998;18(7):3956–65. 9632780 10.1128/mcb.18.7.3956 PMC108980

[55] Kennedy D, French J, Guitard E, Ru K, Tocque B, Mattick J. Characterization of G3BPs: tissue specific expression, chromosomal localisation and rasGAP(120) binding studies. J Cell Biochem. 2001;84(1):173–87. 11746526 10.1002/jcb.1277

[56] Martin S, Zekri L, Metz A, Maurice T, Chebli K, Vignes M, et al Deficiency of G3BP1, the stress granules assembly factor, results in abnormal synaptic plasticity and calcium homeostasis in neurons. J Neurochem. 2013;4(10):12189. 10.1111/jnc.12189 23373770

[57] Matsuki H, Takahashi M, Higuchi M, Makokha GN, Oie M, Fujii M. Both G3BP1 and G3BP2 contribute to stress granule formation. Genes Cells. 2013;18(2):135–46. 10.1111/gtc.12023 23279204

[58] Zekri L, Chebli K, Tourriere H, Nielsen FC, Hansen TV, Rami A, et al Control of fetal growth and neonatal survival by the RasGAP-associated endoribonuclease G3BP. Mol Cell Biol. 2005;25(19):8703–16. 16166649 10.1128/MCB.25.19.8703-8716.2005 PMC1265751

[59] Ortega AD, Willers IM, Sala S, Cuezva JM. Human G3BP1 interacts with beta-F1-ATPase mRNA and inhibits its translation. J Cell Sci. 2010;123(Pt 16):2685–96. 10.1242/jcs.065920 20663914

[60] Winslow S, Leandersson K, Larsson C. Regulation of PMP22 mRNA by G3BP1 affects cell proliferation in breast cancer cells. Mol Cancer. 2013;12(1):1476–4598. 10.1186/1476-4598-12-156 PMC3866477 24321297

