# Supplementary figures and images for: GTPase Activating Protein (Sh3 Domain) Binding Protein 1 Regulates the Processing of MicroRNA-1 during Cardiac Hypertrophy
Source: PLoS One. 2015 Dec 16;10(12):e0145112. doi: 10.1371/journal.pone.0145112 (PMC4684496; doi:10.1371/journal.pone.0145112)

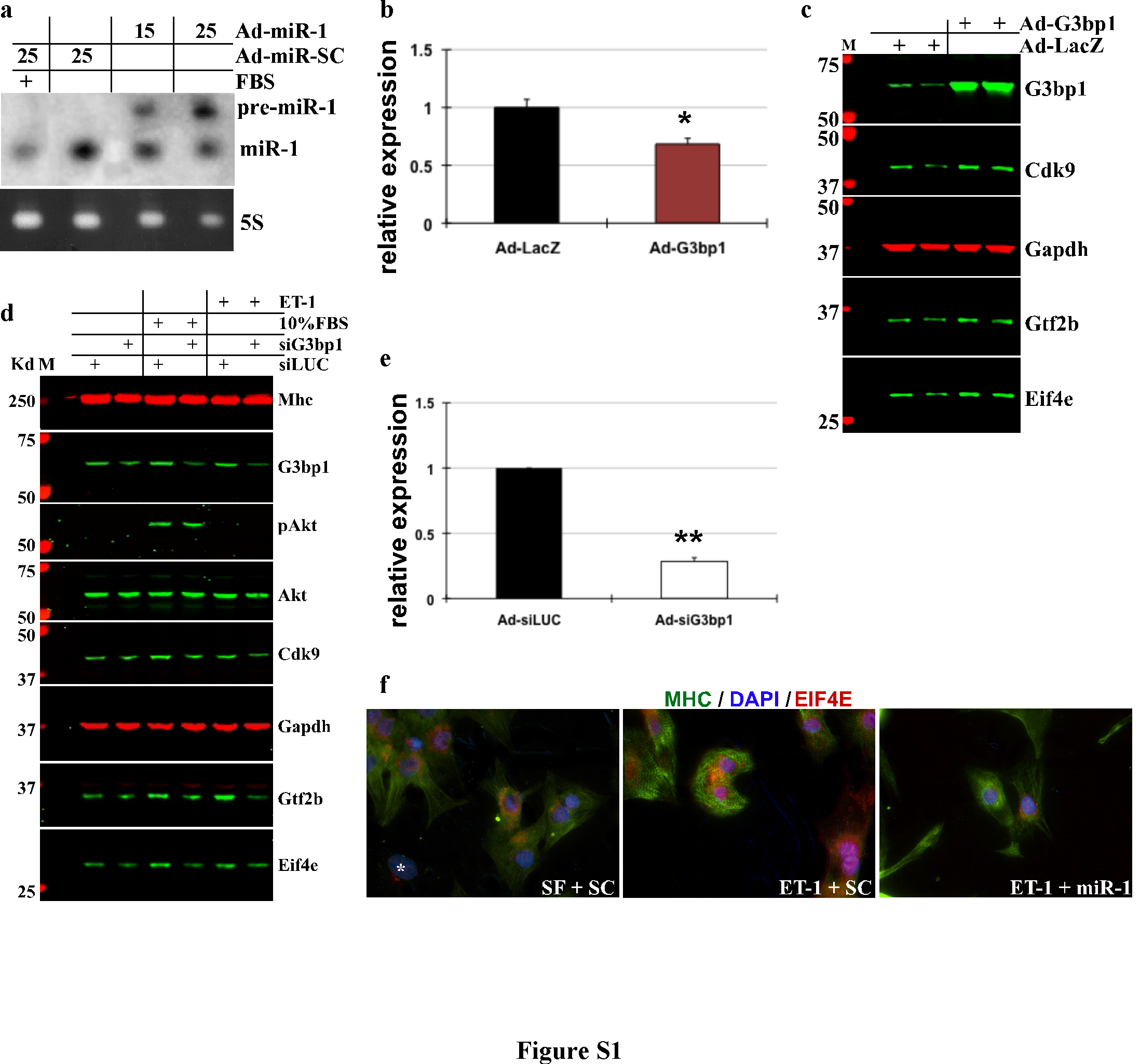

Supplement: S1 File — Figure a. Neonatal myocytes cultured in serum free conditions were infected with increasing doses of Ad-miR-1 for 24hrs, total RNA was extracted and was used for Northern Blot analysis of miR-1 5S is shown as loading control. Figure b. QPCR for miR-1 was performed on total RNA extracted from neonatal myocytes treated with Ad-LacZ or Ad-G3bp1. The graph represents relative miR-1 levels normalized to U6. Error bars represents SEM, and * is p<0.05, n = 3. Figure c. Total protein lysate from neonatal myocytes treated with Ad-LacZ or Ad-G3bp1 was separated by SDS-PAGE and western blotting performed for indicated genes. Figure d. Neonatal myocytes were stimulated with 100nM ET-1 or 10% FBS for 1hr in the presence or absence of Ad-siG3bp1 or Ad-siLUC. Total protein lysate from the cells were separated by SDS-PAGE and western blotting performed for indicated genes. Figure e. QPCR was performed to validate the downregulation of G3bp1 with Ad-siG3bp1 in neonatal myocytes. The graph represents relative G3bp1 mRNA abundance. Error bars represents SEM and ** is p<0.00001, n = 3. Figure f. Immunocytochemistry on neonatal myocytes stimulated with ET-1 in presence of Ad-Sc or Ad-miR-1, and stained for Eif4e, Mhc and Dapi. (TIFF) [file pone.0145112.s002.tiff]

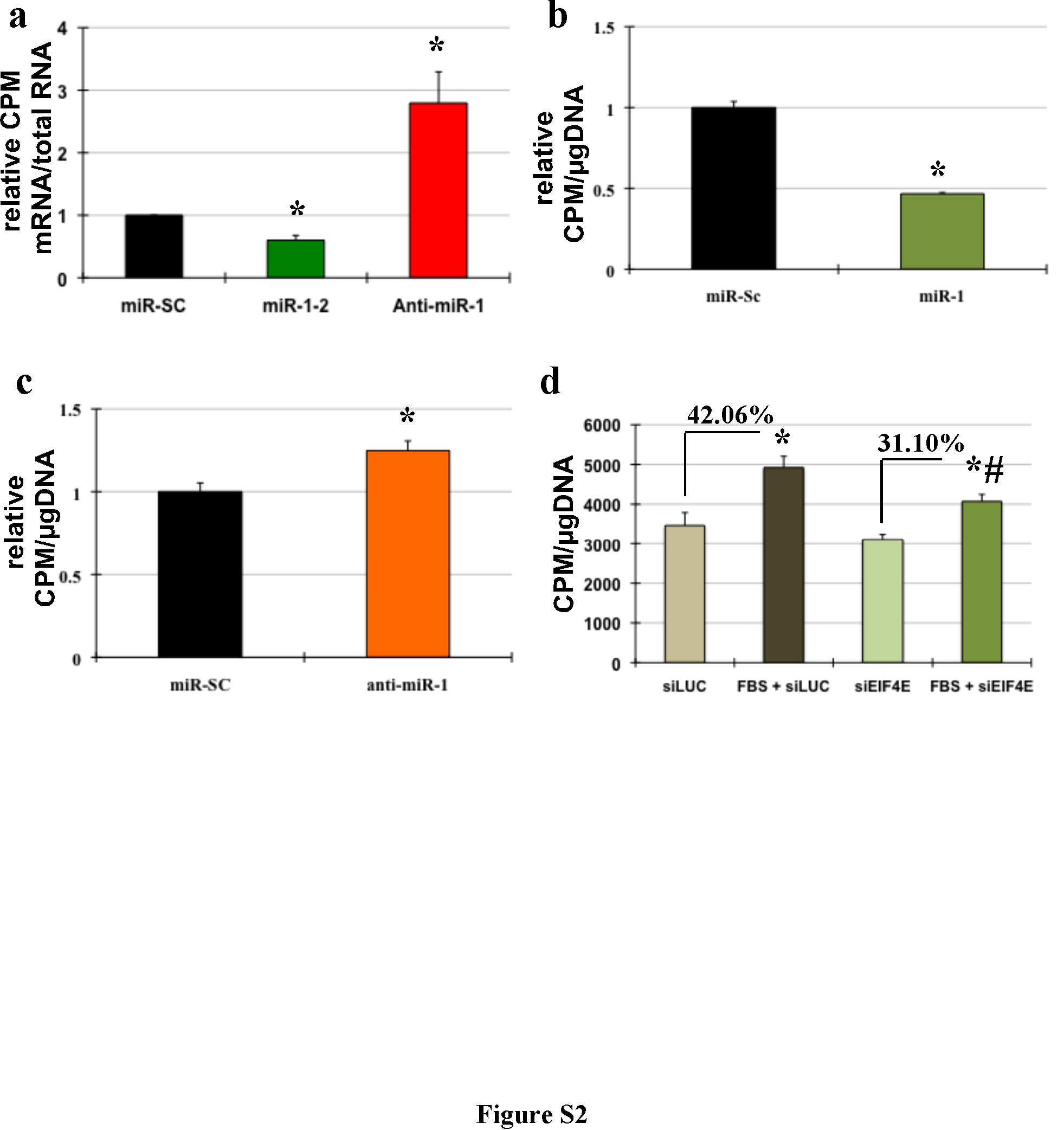

Supplement: S2 File — Figure a. Neonatal myocytes cultured under growth-inhibited conditions were infected with adenoviruses expressing miR-Sc, miR-1 or anti-miR-1 for 24hrs in the presence of [H3]-Uridine before extracting total RNA. mRNA was separated using Oligotex Direct mRNA kit from Qiagen. Counts per minute (CPM) was measured and normalized to CPM of total RNA, averaged and plotted. Error bars represents SEM and * is p<0.05, n = 3. Figure b and Figure c. Neonatal myocytes were infected with miR-Sc, miR-1 or anti-miR-1 as indicated, in the presence of [H3]-leucine. After 24hrs protein and DNA was extracted and [H3]-leucine incorporation measured by a scintillation counter. CPM was measured and normalized to DNA, averaged and plotted. Error bars represents SEM and * is p<0.05, n = 3. Figure d. Neonatal myocytes cultured in growth-inhibited conditions were treated with siLUC or siEif4e. After 24hrs, cells were supplemented with [H3]-leucine and 10% fetal bovine serum for additional 24hrs. Protein and DNA was precipitated and extracted as per protocol, and incorporation of [H3]-leucine measured and plotted as described above. Error bars represents SEM and * is p<0.05 vs. their respective controls. # is p<0.01 siEif4e with FBS vs. siLUC with FBS. The graph also indicates the percent increase in protein synthesis after FBS treatment in cardiomyocytes after treatments with siLUC or siEif4e, as indicated. (TIFF) [file pone.0145112.s003.tiff]
